# Quantification of Biventricular Myocardial Strain Using CMR Feature Tracking: Reproducibility in Small Animals

**DOI:** 10.1155/2021/8492705

**Published:** 2021-01-22

**Authors:** Hao Li, Yangyang Qu, Patrick Metze, Florian Sommerfeld, Steffen Just, Alireza Abaei, Volker Rasche

**Affiliations:** ^1^Core Facility Small Animal Imaging, Ulm University, Ulm, Germany; ^2^Department of Internal Medicine II, Ulm University Medical Center, Ulm, Germany

## Abstract

Myocardial strain is a well-validated parameter for evaluating myocardial contraction. Cardiovascular magnetic resonance myocardial feature tracking (CMR-FT) is a novel method for the quantitative measurements of myocardial strain from routine cine acquisitions. In this study, we investigated the influence of temporal resolution on tracking accuracy of CMR-FT and the intraobserver, interobserver, and interstudy reproducibilities for biventricular strain analysis in mice from self-gated CMR at 11.7 T. 12 constitutive nexilin knockout (Nexn-KO) mice, heterozygous (Het, *N* = 6) and wild-type (WT, *N* = 6), were measured with a well-established self-gating sequence twice within two weeks. CMR-FT measures of biventricular global and segmental strain parameters were derived. Interstudy, intraobserver, and interobserver reproducibilities were investigated. For the assessment of the impact of the temporal resolution for the outcome in CMR-FT, highly oversampled semi-4 chamber and midventricular short-axis data were acquired and reconstructed with 10 to 80 phases per cardiac cycle. A generally reduced biventricular myocardial strain was observed in Nexn-KO Het mice. Excellent intraobserver and interobserver reproducibility was achieved in all global strains (ICC range from 0.76 to 0.99), where global right ventricle circumferential strain (RCS_SAX_) showed an only good interobserver reproducibility (ICC 0.65, 0.11-0.89). For interstudy reproducibility, left ventricle longitudinal strain (LLS_LAX_) was the most reproducible measure of strain (ICC 0.90, 0.71-0.97). The left ventricle radial strain (LRS_SAX_) (ICC 0.50, 0.10-0.83) showed fair reproducibility and RCS_SAX_ (ICC 0.36, 0.14-0.74) showed only poor reproducibility. In general, compared with global strains, the segmental strains showed relatively lower reproducibility. A minimal temporal resolution of 20 phases per cardiac cycle appeared sufficient for CMR-FT strain analysis. The analysis of myocardial strain from high-resolution self-gated cine images by CMR-FT provides a highly reproducible method for assessing myocardial contraction in small rodent animals. Especially, global LV longitudinal and circumferential strain revealed excellent reproducibility of intra- and interobserver and interstudy measurements.

## 1. Introduction

Quantitative assessment of cardiac contractile function is an essential component of cardiac imaging study. Ejection fraction (EF) is the most widely used global parameter to describe cardiac function. However, its volumetric nature, suboptimal reproducibility, and deficiency to reflect regional cardiac function limit its application to detect subtle changes in cardiac function and provide detailed information on myocardial mechanical activity [1, 2].

Myocardial strain is defined as the percentage change of myocardial fiber length compared with the initial state in a certain direction by an internal or external force and is assumed a more in-depth characterization of cardiac function [1, 3]. Strain is a vector with distinctive spatial orientations of myocardial contraction and alters early in disease pathogenesis and varies with cardiac pathologies [4–6]. According to the different directions in which the myocardium deforms, imaging modalities calculate myocardial strain in three principal directions (radial, circumferential, and longitudinal). Over the years, cardiovascular magnetic resonance (CMR) imaging has proven to be an accurate and versatile imaging modality to quantify myocardial deformation due to its feasibility to provide high-quality images of the heart with high temporal and spatial resolution, permitting accurate myocardial border delineation and cardiac assessment [7–10].

With the introduction of the myocardial tagging technique by Zerhouni et al. and Axel and Dougherty [11, 12], the measurement of myocardial strain using CMR imaging became possible. In tagging MRI, a pulse train combined with a gradient pulse is applied to create saturated stripes and spatially modulate the longitudinal magnetization prior to the conventional image acquisition [10]. The measurement of myocardial strain is based on the utilization of tag deformation over the cardiac cycle [13]. However, the requirement for additional image acquisition sequences and time-consuming postprocessing limits its widespread application.

Recently, a relatively novel 2D imaging technique, CMR feature tracking (CMR-FT), was introduced to calculate global and segmental myocardial strain using standard balanced steady-state free precession (b-SSFP) sequence without the requirement for dedicated acquisition [8]. The CMR-FT algorithm is mainly based on optical flow technology and was commercially available. After defining regions of endocardial and epicardial borders at end-diastole, CMR-FT tracks the respective features over the cardiac cycle by correlation of similar regions in the subsequent frames [10, 14]. As CMR-FT is a promising novel method for the quantification of myocardial strain from routinely acquired cine CMR images without specific strain image acquisition sequence, and the analysis can be performed in a retrospective manner without time-consuming postprocessing, it has the potential for a fast assessment of myocardial deformation. CMR-FT has been clinically validated against myocardial tagging technique [7, 15], and excellent inter and intraobserver agreement and high interstudy reproducibility were reported [16–18]. However, only one preliminary study was reported in preclinical research by Lapinskas et al. [9], they reported a high inter- and intraobserver reproducibility in left ventricle global circumferential and longitudinal strain in healthy mice, whereas reproducibility of radial strain was limited. However, only a few wild-type animals without any cardiovascular pathology were enrolled, thus limiting the generalization of the obtained results. And relatively low temporal resolution (15 phases per cardiac cycle) cine images were applied; the accurate interpretation of the results was concerned.

This study is aimed at evaluating the minimally required temporal resolution to ensure diagnostic accuracy and reproducibility of biventricular strain analysis in nexilin knockout (Nexn-KO) induced heart failure mice [19]. The interobserver, intraobserver, and interstudy reproducibilities of CMR-FT were investigated.

## 2. Methods

### 2.1. Animals

12 constitutive heterozygous (Het, *N* = 6: 3 males, 3 females) and wild-type (WT, *N* = 6: 3 males, 3 females) Nexn KO mice were included in this study [19]. Mice were housed under standard conditions with ad libitum access to water and food.

Animal experiments were approved by the regional board of Tübingen and conducted according to German law for the welfare of animals and regulations for the care and use of laboratory animals. All institutional and national guidelines for the care and use of laboratory animals were followed and approved by the appropriate institutional committees.

### 2.2. CMR Image Acquisition

Experiments were performed on an 11.7 Tesla MRI system dedicated to small animal applications (BioSpec 117/16, Bruker BioSpin, Ettlingen, Germany) equipped with a high-performance gradient system (B-GA9S HP, Bruker BioSpin, Ettlingen, Germany) providing a maximal gradient strength of 760 mT/m with maximal slew rate of 6840 T/m/s. A 72 mm quadrature transmit/receive coil was used for radiofrequency transmission in conjunction with a dedicated four-element thorax coil (RAPID Biomedical, Rimpar, Germany) for data acquisition.

All animals were anesthetized in an induction container attached to the vaporizer by exposure to 5% isoflurane. The isoflurane was then maintained between 1.0 and 1.5% in medical air (0.1 L/min) during the entire procedure to maintain the respiratory frequency between 60 and 80 respiratory cycles per minute [20]. Mice were placed in a prone position, and the respiratory rate was monitored with a balloon pressure sensor. Examinations were performed in a temperature-controlled setting, and the temperature of the mice was maintained by a water heating blanket. The fluctuation of rectal temperature was kept below 1°C.

CMR cine data were acquired by applying a conventional Cartesian self-gated sequence (IntraGate©, ParaVision 6.0.1, Bruker BioSpin, Ettlingen, Germany) with Sensitivity Encoding (SENSE) acceleration [20, 21]. Slice planning was performed as suggested earlier [21] ensuring high reproducibility of the image geometry for subsequent examinations. For cardiac planning, three-plane cardiac scout images were obtained using a nongated fast gradient echo imaging sequence, followed by a semi-2 chamber (2CH), semi-4 chamber (4CH), and short-axis (SAX) images subsequently. Then, two long-axis cine scans in 2CH and 4CH were accurately planed and acquired using retrospectively gated Fast Low Angle Shot (FLASH) imaging approach. The cine 2/4CH images were used to plan the subsequent stack of cine SAX images. The number of short-axis slices was adjusted to ensure full coverage of the left and right ventricles in end-diastole. The following parameters were set [20]: echo/repetition time TE/TR = 1.04/5.62 ms, flip angle *α* = 15°, matrix size = 256 × 256, in-plane resolution Δ*r* = 0.1172 mm^2^, slice thickness *s*_D_ = 1.0 mm, bandwidth bw = 125 kHz, 20 phases per cardiac cycle, 200-fold oversampling, acceleration factor = 2, and acquisition time *T*_ACQ_ = 2 m 37 s per slice.

For the interstudy reproducibility assessment, all mice were rescanned with the same protocol within two weeks.

For the investigation of the influence of temporal resolution on the resulting strain values, additional Cine-4CH and midventricular SAX images were acquired with a similar MR protocol but higher 600-fold oversampling, resulting in an acquisition time *T*_ACQ_ = 7 min 52 s per slice. Cine data with 10, 15, 20, 30, 40, 60, and 80 phases per cardiac cycle were reconstructed with the vendors SW (IntraGate©, ParaVision 6.0.1, Bruker BioSpin, Ettlingen, Germany).

### 2.3. Data Analysis

#### 2.3.1. Biventricular Volumes and Function

Biventricular functional parameters and myocardial deformation were analyzed using Segment v3.0 R7912 (http://segment.heiberg.se) [22]. Biventricular end-diastolic volume (EDV), end-systolic volume (ESV), stroke volume (SV), ejection fraction (EF), left ventricle mass at end-diastole (LVMED), and left ventricle mass at end-systole (LVMES) were quantified manually by an experienced physician and the cardiac output (CO) calculated.

#### 2.3.2. Feature Tracking

For CMR feature tracking, interframe deformation fields derived from nonrigid registration between cardiac frames are analyzed for calculation of myocardial strain curves [22]. To guide the tracking, an initial segmentation in the end-diastolic frame has to be performed for all slices, which acts as the reference frame for subsequent strain calculation. Further, a geometrical reference point in the middle of the septum has to be identified for guiding the segmental analysis using the American Heart Association (AHA) 16-segment approach [22, 23]. The border between LV and RV is automatically matched. During tracking, the contours are propagated and registered automatically to the subsequent time frames. Respective deformation fields are derived, and the myocardial strain and strain rate curves are calculated.

For this study, global and segmental LV longitudinal (LLS_LAX_), circumferential (LCS_SAX_), and radial strain (LRS_SAX_), as well as RV longitudinal (RLS_LAX_) and circumferential strain (RCS_SAX_), were calculated from 4 chamber long-axis and short-axis images. The intraobserver variability assessment was performed in all mice by analyzing the data twice within a time interval of 2 weeks (H.L.). For the assessment of the interobserver reliability, analysis was done by 2 experienced, independent observers (H.L. and F.S.) and the interstudy reproducibility was assessed from the repeated measurements by an experienced reader (H.L.).

### 2.4. Statistical Analysis

All image analyses were done blindly, and data were presented as the mean ± standard deviation (SD). Data was analyzed using SPSS 25.0 (IBM, USA) and MedCalc 19.1.3 (MedCalc Software, Belgium). The comparisons of biventricular functional parameters and myocardial deformation between the two groups were assessed by an unpaired Mann–Whitney *U* test, with *p* values below 0.05 being considered significant. The inter- and intraobserver reproducibility and interstudy reproducibility were assessed using the intraclass correlation coefficient (ICC), coefficient of variations (CoVs), and Bland-Altman analysis. Agreement was considered excellent for ICC > 0.74, good for ICC 0.6-0.74, fair for ICC 0.40-0.59, and poor for ICC < 0.40. Linear regression analysis was performed for intraobserver, interobserver, and interstudy correlation of myocardial strain. A Friedman test was applied for investigating the impact of temporal resolution on biventricular strain analysis.

## 3. Results

**Figure 1:**
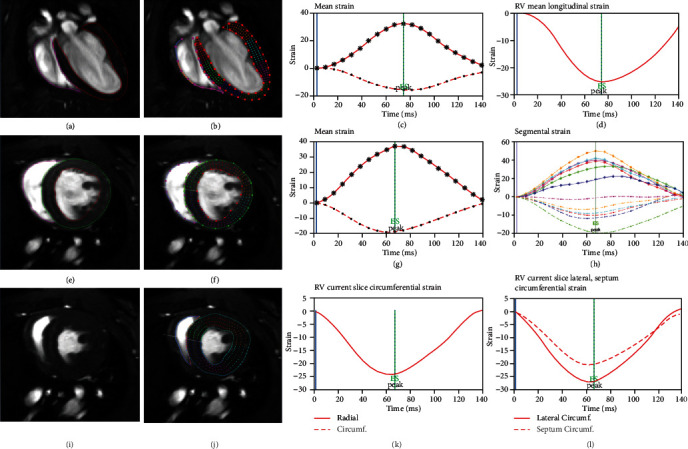
High-resolution cine CMR images with biventricular segmentation and CMR-FT myocardial strain curves in mouse. End-diastolic frame of semi-4CH (a) and the following image after application of feature tracking algorithm (b). The LV (c) and RV (d) strain curves and peak strain obtained from semi-4CH cine images. End-diastolic (e) and end-systolic (i) frame of mid-SAX images and the following tracking pattern of end-diastolic image (f) and end-systolic image (j). The global strain curves for LV (g) and RV (k) and the segmental strain curves for LV (h) and RV (l) derived from mid-SAX cine images. LV: left ventricle; RV: right ventricle; CMR-FT: cardiovascular magnetic resonance feature tracking; 4CH: 4 chamber; SAX: short axis.

Using the described self-gated CMR imaging protocols, high-quality images were obtained in all animals (Figure 1). In all cases, the respiratory rate could be maintained between 60 and 80 cycles per minute and changes of the recorded temperature were below ±0.5°C.

### 3.1. Baseline Characteristics

Biventricular volumetric and functional parameters of study subjects are shown in Table 1. Mean heart rates were 454 ± 33 vs. 393 ± 48 for Nexn-WT and Nexn-Het, respectively (*p* = 0.07). Compared with WT, the left ventricular EDV and ESV resulted in markedly higher in the Het Nexn-KO mice (*p* < 0.001), resulting in a relatively lower EF at rest (*p* < 0.0001). No statistically significant difference for LVM at end-diastole and end-systole, and SV were observed between the two groups (*p* > 0.05). A slightly higher CO was observed in WT mice, however without statistical significance (WT, 19.91 ± 3.07 ml/min vs. 16.92 ± 1.66 ml/min, Het; *p* = 0.06). For the right ventricle, a relatively higher ESV (WT, 12.85 ± 3.03 *μ*l vs. 19.35 ± 4.21 *μ*l, Het; *p* < 0.05) and lower EF (WT, 77.64 ± 2.79% vs. 67.95 ± 3.41%, Het; *p* < 0.001) were observed in Het mice. No significant differences were observed in EDV (WT, 56.95 ± 8.40 *μ*l vs. 59.89 ± 8.11 *μ*l, Het; *p* = 0.55) and SV (WT, 44.10 ± 5.82 *μ*l vs. 40.54 ± 4.46 *μ*l, Het; *p* = 0.26).

### 3.2. Biventricular Strain

**Figure 2:**
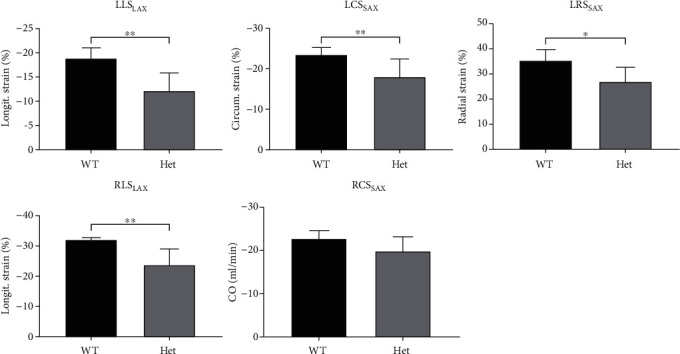
Comparison of biventricular myocardial strain of CMR-FT between nexilin knockout heterozygous (Het) and wild-type (WT) mice. LLS_LAX_: left ventricular long-axis longitudinal strain; LCS_SAX_: left ventricular short-axis circumferential strain; LRS_SAX_: left ventricular short-axis radial strain; RLS_LAX_: right ventricular long-axis longitudinal strain; RCS_SAX_: right ventricular short-axis circumferential strain. ^∗^*p* < 0.05 and ^∗∗^*p* < 0.01.

The mean peak global biventricular strains are provided in Figure 2. Compared with the Nexn Het group, significantly higher LLS_LAX_ (WT, −18.80 ± 2.09% vs. −12.13 ± 3.70%, Het; *p* < 0.01), LCS_SAX_ (WT, −23.47 ± 1.84% vs. −18.01 ± 3.23%, Het; *p* < 0.01), LRS_LAX_ (WT, 34.95 ± 4.42% vs. 26.88 ± 5.80%, Het; *p* < 0.05), and RLS_LAX_ (WT, −31.67 ± 0.96% vs. −23.66 ± 5.07%, Het; *p* < 0.01) were observed in the WT group. Slightly higher RCS_SAX_ was observed in the WT group, but without significant difference (WT, −22.45 ± 1.95% vs. −19.85 ± 3.23%, Het; *p* = 0.13).

### 3.3. Intraobserver, Interobserver, and Interstudy Variability of CMR-FT

**Figure 3:**
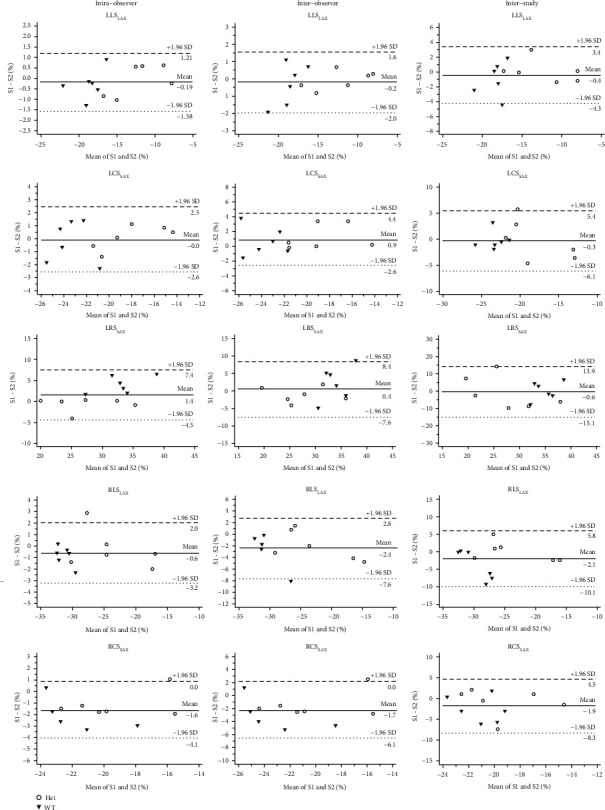
Bland-Altman plots with limits of agreement (1.96 standard deviations) demonstrate the intraobserver, interobserver, and interstudy reproducibilities (S1 and S2) of CMR-FT for LV and RV strain assessments. The relative variability is defined as the difference between observations divided by their mean. LLS_LAX_: left ventricular long-axis longitudinal strain; LCS_SAX_: left ventricular short-axis circumferential strain; LRS_SAX_: left ventricular short-axis radial strain; RLS_LAX_: right ventricular long-axis longitudinal strain; RCS_SAX_: right ventricular short-axis circumferential strain.

**Figure 4:**
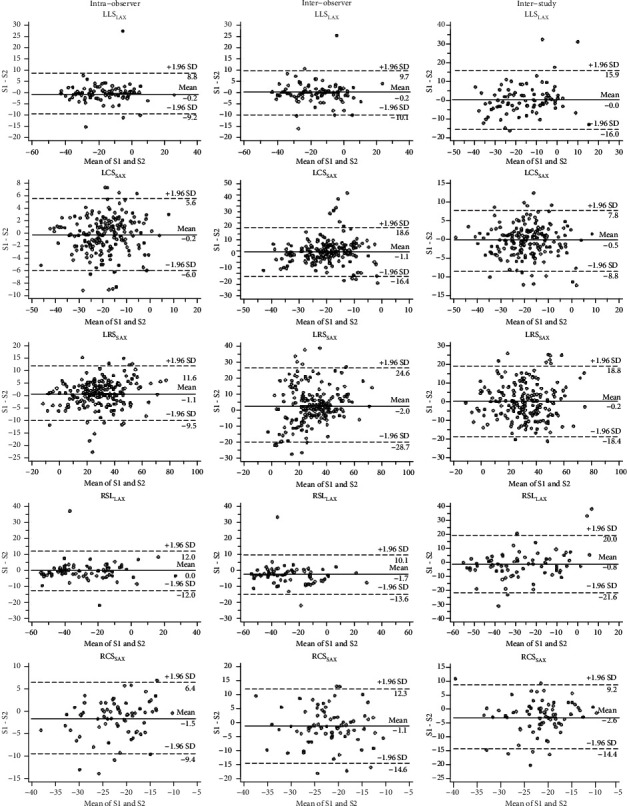
Bland-Altman plots with limits of agreement (1.96 standard deviations) demonstrate the intraobserver, interobserver, and interstudy reproducibilities (S1 and S2) of CMR-FT for segmental LV and RV strain assessments. The relative variability is defined as the difference between observations. LLS_LAX_: left ventricular long-axis longitudinal strain; LCS_SAX_: left ventricular short-axis circumferential strain; LRS_SAX_: left ventricular short-axis radial strain; RLS_LAX_: right ventricular long-axis longitudinal strain; RCS_SAX_: right ventricular short-axis circumferential strain.

The mean difference, limits of agreement, ICC, and CoVs, as well as the Bland-Altman analysis, are summarized for global (Table 2 and Figure 3) and segmental (Table 3 and Figure 4) strain parameters. Excellent reproducibility could be observed in global LV myocardial strain intra- and interobserver variability (ICC > 0.74). Regarding interstudy reproducibility, excellent variability for LLS_LAX_ (ICC 0.90, 0.71-0.97) and LCS_SAX_ (ICC 0.77, 0.38-0.93), but only fair variability for LRS_LAX_ (ICC 0.5, 0.10-0.83), was observed. For the RV, RLS_LAX_ showed excellent intraobserver (ICC 0.97, 0.89-0.99) and interobserver (ICC 0.84, 0.30-0.96), and good interstudy (ICC 0.50, 0.1-0.83) reproducibility. With ICC 0.78 (0.02-0.95), 0.65 (0.11-0.89) and ICC 0.36 (0.14-0.74) for intra- and interobserver, and interstudy level, respectively, RCS_SAX_ showed least reproducibility.

For the segmental strain reproducibility, all strain parameters showed good to excellent intraobserver reproducibility. For the interobserver variability, longitudinal strains were the most reproducible parameters, with ICC 0.93, CoVs 19.81% for left ventricle and ICC 0.95, CoVs 19.5% for right ventricle, respectively. However, only poor to fair reproducibility was observed for circumferential and radial strains, with ICC range from 0.47-0.64, CoVs range from 18.35%-42.6%. For the interstudy reproducibility, biventricular longitudinal and left ventricle circumferential strains showed good reproducibility (ICC range from 0.80-0.91); the left ventricle radial (ICC 0.84, CoVs 58.3%) and right ventricle circumferential (ICC 0.54, CoVs 15.29%) strains only showed poor and fair reproducibility.

### 3.4. Linear Regression Analysis

**Figure 5:**
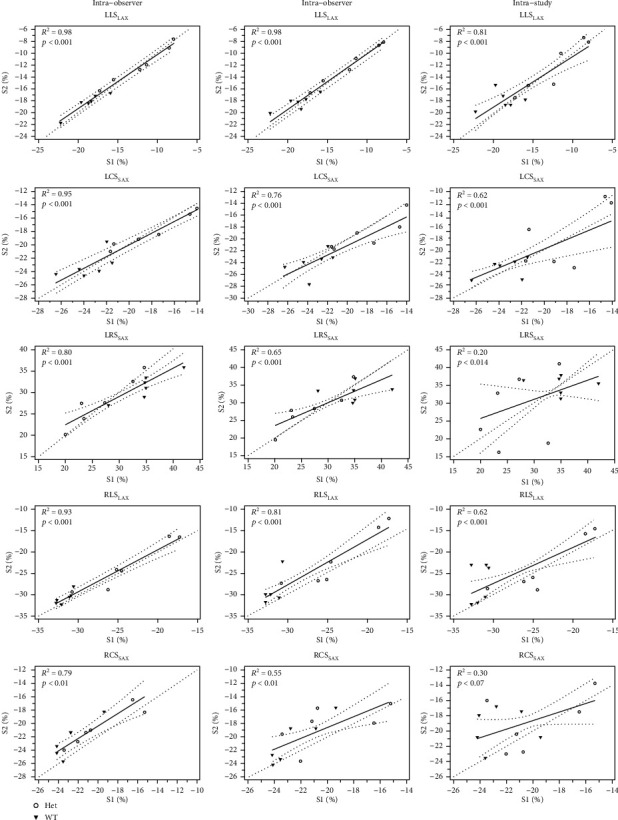
Scatterplots showing the intraobserver, interobserver, and interstudy correlations between two readings (S1 and S2) for global biventricular myocardial strain of CMR-FT. Our linear regression analysis shows an excellent correlation for all LV myocardial strain values, except LRS_SAX_ in interstudy measurement. The reproducibility of RV is variable; RCS_SAX_ is the least reproducible myocardial strain value. The regression line is given with a 95% confidence interval. *R*-squares and *p* values are reported in the left upper corner of each graph. LLS_LAX_: left ventricular long-axis longitudinal strain; LCS_SAX_: left ventricular short-axis circumferential strain; LRS_SAX_: left ventricular short-axis radial strain; RLS_LAX_: right ventricular long-axis longitudinal strain; RCS_SAX_: right ventricular short-axis circumferential strain.

Linear regression analysis for all mice revealed an adequate intra- and interobserver, and interstudy correlation for global LLS_LAX_, LCS_SAX_, and RLS_LAX_ with *R*-square ranging between 0.62 and 0.98. The global LRS_LAX_ and RCS_SAX_ showed good intraobserver and fair interobserver correlation, but only poor interstudy correlation (*R*-square 0.20 to 0.30). The scatter plots with individual regression line, 95% confidence interval, *R*-square, and corresponding *p* values are provided in Figure 5.

### 3.5. Effects of Temporal Resolution

**Figure 6:**
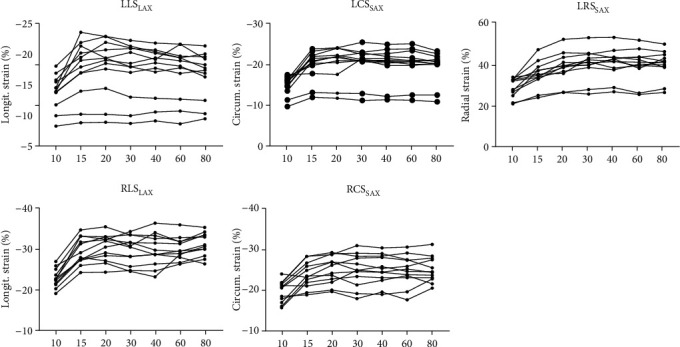
Influence of temporal resolution on feature tracking accuracy of biventricular strain measurement. Individual cases are shown with temporal resolution range from 10 frames to 80 frames per cardiac cycle. LLS_LAX_: left ventricular long-axis longitudinal strain; LCS_SAX_: left ventricular short-axis circumferential strain; LRS_SAX_: left ventricular short-axis radial strain; RLS_LAX_: right ventricular long-axis longitudinal strain; RCS_SAX_: right ventricular short-axis circumferential strain.

Descriptive values (mean ± SD) and results of statistical analysis of all the biventricular myocardial strain parameters are provided in Table 4 and Figure 6. The signal-to-noise ratio in the myocardium (SNR_m_) and the LV cavity (SNR_b_) and the contrast-to-noise ratio (CNR) between the myocardium and LV cavity for different temporal resolutions are shown in Supplementary Material S1. As expected, SNR clearly drops with increasing temporal resolution. Significantly decreased myocardial strain was observed with 10 phases per cardiac cycle (*p* < 0.01). Regarding temporal resolution of 15 phases per cardiac cycle, significantly decreased LRS_SAX_ (*p* < 0.001) could be observed. With increased temporal resolution range from 20 to 80 phases per cardiac cycle, biventricular myocardial strain did not show any significant difference (*p* > 0.05). Examples of qualitative global and segmental biventricular strain curve comparison between different temporal resolutions are shown in Supplementary Materials S2–S5.

## 4. Discussion

The present study was conducted to validate biventricular myocardial strain analyzed by CMR-FT and to determine its intra- and interobserver and interstudy reliability in a nexilin-induced heart failure mouse model. In addition, the impact of temporal resolution on the tracking accuracy of CMR-FT was assessed. Overall, CMR-FT showed promising results for the quantitative evaluation of biventricular myocardial deformation. Intra- and interobserver and interstudy variability in terms of CoVs, ICC, and Bland-Altman yielded modest to excellent results for global LV myocardial strain and RV longitudinal strain. However, global RV circumferential strain and LV radial strain yielded only poor interstudy reproducibility. Compared with global strain, the segmental strain showed relatively lower interobserver and interstudy reproducibility with best performance for longitudinal strain. A minimal temporal resolution of 20 phases per cardiac cycle appeared sufficient for strain analysis.

### 4.1. CMR Feature Tracking

Evaluation of cardiac contractile performance is crucial in practice to assess disease severity and monitor therapeutic progression. It is well recognized that global measures, such as ejection fraction, are not sufficient to provide a comprehensive evaluation of cardiac mechanics due to the complex architectural arrangement of myofibers [2]. Approximately half of the heart failure patients do not show markedly abnormal but preserved ejection fraction [24]. Cardiac strain has been introduced as a sensitive measure of myocardial deformation, enabling evaluation of different spatial components of contractile function in three principal directions (radial, circumferential, and longitudinal).

Feature tracking is a relatively novel 2D tissue tracking technique. After Maret et al. [8] investigated strain analysis with cine balanced steady-state free precession (b-SSFP) sequence on scar patients, feature tracking gained great interest by allowing measurement of myocardial deformation without the requirement for dedicated acquisition and postprocessing [25, 26]. The CMR-FT algorithm is based on optical flow methods, and it uses the ventricular boundaries to track the patterns of features or irregularities in the successive time frames [27]. Based on this technology, multiple commercial strain analysis software packages have been introduced [1]. Taylor et al. [28] and Mangion et al. [26] reported normal values for LV myocardial strain measurements using FT-CMR in healthy individuals. Later, Pryds et al. [25] evaluated the performance of offline CMR-FT analysis for LV myocardial deformation assessment in patients with a variety of cardiovascular disease and healthy subjects, then further compared with speckle tracking echocardiography (STE). However, only modest correlation was observed between CMR-FT and STE. Liu et al. [29] reported the RV longitudinal strain with CMR-FT in healthy subjects. Later, Stiermaier et al. [30] investigated RV strain assessment in Takotsubo syndrome patients to optimize risk stratification. These studies confirmed its feasibility of biventricular strain assessment in clinical research.

In preclinical research, small rodent animal models of human cardiovascular disease have proved to be tremendously valuable laboratory tools to investigate the basic underlying mechanism of normal and abnormal cardiac function because of the development of genetically engineered mice and their short reproductive cycle. Hence, investigating the feasibility of CMR-FT in small animals can give further insight information of cardiac disease. Lapinskas et al. [9] investigated CMR-FT in healthy mice with a 3 Tesla small animal MRI scanner. However, they only performed rather low temporal resolution (15 phases per cardiac cycle) cine images. The validation in cardiovascular disease and tracking accuracy of minimal temporal resolution need further investigation. Moreover, due to the small size of the mouse heart (5-6 mm left ventricle diameter, approximately 0.2 g of heart weight) [31], even though the lower signal-to-noise ratio (SNR) can partly be compensated with dedicated coils at 1.5 T and 3 T scanner [32, 33], ultra-high-field MRI (>7 Tesla) is most commonly used in small animal CMR imaging to provide images with sufficient spatial and temporal resolution. In our study, we have used a nonrigid, elastic image registration algorithm available from Segment (Medviso AB, Sweden) to test its feasibility of quantitative assessment of biventricular myocardial strain in nexilin knockout mice at 11.7 Tesla. Mutations in the gene encoding nexilin are associated with cardiomyopathy, especially for the dilated cardiomyopathy (DCM), and resulting in impaired cardiac contraction [19, 34, 35]. In our study, a clear decreased biventricular myocardial strain, except RCS_SAX_ (WT, −22.45 ± 1.95% vs. −19.85 ± 3.23%, Het; *p* = 0.13), could be observed in the heterozygous group (*p* < 0.05) supporting the outcome of the volumetric and functional characteristics.

### 4.2. Reproducibility of Myocardial Deformation Analysis Using CMR-FT

In cardiovascular imaging, it is particularly important to ensure high reproducibility within and between observers and between different acquisitions to ensure sensitive quantification of in vivo data. 3D self-gated FLASH may show more consistent strain values over the volume since all data are acquired simultaneously, and thus, strain values should be less effected by heart rate variation which may occur in case of subsequent sampling of 2D slices as done with 2D self-gated FLASH. However, 3D FLASH techniques suffer from long acquisition times and artifacts, which may limit its widespread applications, such as pharmacological stress imaging and unstable animals with cardiovascular disease. Here, the 2D self-gated FLASH sequence is a more flexible and convenient acquisition technique for feature tracking strain analysis, and has proven to be a valuable technique with excellent reproducibility for the functional imaging [21, 36],

High reproducibility for LV and RV global longitudinal and circumferential strains was proved in clinical research, and global strain assessment was more reproducible than segmental analysis [17, 22, 26, 29, 37]. In preclinical research, Lapinskas et al. [9] reported excellent inter- and intraobserver reproducibility of CMR-FT for global circumferential and longitudinal strain in healthy mice, whereas reproducibility of radial strain was weak. In our study, with the high spatial and adequate temporal resolution images, excellent intra- and interobserver reproducibility was achieved in all global parameters (ICC range from 0.76 to 0.99); only RCS_SAX_ showed a good reproducibility. For interstudy reproducibility, global LLS_LAX_ was the most reproducible measure of strain (ICC 0.90, 0.71-0.97). The global LRS_SAX_ (ICC 0.50, 0.10-0.83) showed fair and global RCS_SAX_ (ICC 0.36, 0.14-0.74) showed poor interstudy reproducibility. The results were highly identical to regression analysis. In the contrast, the segmental strain showed great intraobserver reproducibility. However, the interobserver and interstudy reproducibilities were worse than for global strains. Compared with the left ventricle, the right ventricle is much more geometrically complex. The walls are much thinner, and there are more and complex trabeculation, leading to challenges for RV quantification. In contrast to human studies, achieving high interstudy reproducibility in small animal imaging appears more challenging. As anesthesia is generally required in small animal imaging to provide immobility, the effects on cardiac function are generally of concern. Moreover, circadian rhythms and body temperature also impact cardiac function. In our experience, inhalation anesthesia with isoflurane was used, since they provide greater safety, lesser cardiovascular depression, and rapid recovery and convenient adjustments and maintenance during scan. Further body temperature control of the animal and scan time seems mandatory to avoid deterioration of the derived functional parameters.

### 4.3. Effects of Temporal Resolution

Both spatial and temporal resolutions are crucial to ensure accurate quantification of myocardial deformation. Especially for small rodent animal imaging, the small organ volume and high heart rate rise concerns. With the self-gating protocol, high spatial resolution with sufficient SNR and contrast-to-noise ratio (CNR) cine images was provided. Too low temporal resolution will miss phases of rapid volume changes and may lead to underestimation of displacement and myocardial strain. Moreover, due to larger displacements between images, image decorrelation will impact the accuracy of the tracked feature patterns in the subsequent frames [27]. The minimal temporal resolution for ensuring sufficient data fidelity in CMR-FT myocardial strain analysis has not been evaluated. In clinical routine, the temporal resolution of the different modalities is variable. Normally, 25-35 phases per cardiac cycle are preferred for CMR-FT [1]. In our case, we investigated the impact of temporal resolution on strain measurement of CMR-FT on small animal imaging. Strain analysis from cine data reconstructed with different temporal resolutions (10 to 80 frames per cardiac cycle) revealed reduced biventricular strain values with 10 frames per cardiac cycle and decreased LRS_SAX_ with 15 frames per cardiac cycle, but no significant differences for 20 and more frames, thus recommending a minimal number of 20 cardiac phases over the cardiac cycle for accurate strain quantification. Moreover, due to the short isovolumetric contraction phase, a plateau forming of LV myocardial strain curve could be observed with temporal resolution of 60 frames per cardiac cycle in Supplementary Materials S2-S3, and more obvious for 80 frames per cardiac cycle.

### 4.4. Comparison with Speckle Tracking

STE is a comparable and more established technique and has been widely applied in both clinical and preclinical routines [38, 39]. In STE, myocardial motion and deformations are tracked by natural acoustic reflections and interference patterns in speckles within an ultrasonic window [40]. The image-processing algorithm tracks defined regions of interest, which requires a high frame rate (>200 frames/s) during image acquisition and high spatial resolution to obtain quality speckle tracking information [41]. Previous studies have shown the preclinical utility of speckle tracking for LV contractility evaluation in multiple cardiovascular diseases [39, 42, 43]. With speckle-tracking-based strain analysis, Zhou et al. [42] monitored early-stage changes in average global radial and longitudinal strain in the heart of type I diabetic Akita mice at 12 weeks of age, and Li et al. [44] reported reduced radial and circumferential strain in db/db type II diabetic mice when there was no appreciable reduction in global LV function.

Many clinical studies have compared myocardial strain assessment of CMR-FT and STE in patients with various cardiac diseases. Obokata et al. [45] reported good global longitudinal strain and excellent global circumferential strain, but only fair global radial strain correlation between CMR-FT and 2D/3D STE. Only modest correlation and agreement was observed between CMR-FT and STE by Pryds et al. [25]. Maybe further studies with larger patient cohorts need to be performed. Comparative studies in preclinical studies have not yet been performed. Compared with other STE studies [42, 43, 46], our CMR-FT-derived myocardial strain values of nexilin wild-type mice are in excellent concordance with the control groups. However, the direct comparison of STE and CMR-FT in small animals needs further investigation. Lapinskas et al. [9] reported much lower CMR-FT-derived LV myocardial strain values of healthy mice, especially for LV radial strain. It might be attributed to lower temporal resolution and image quality. Further, the influence of different software vendors must be considered. Both techniques contain advantages and disadvantages. STE is convenient to perform and largely available. However, the STE technique may be affected by limited acoustic windows, and relatively worse image quality in the distal part of the ultrasound sector may lead to inconsistency of accuracy and reproducibility, especially for segmental measurements [27]. Conversely, CMR imaging is time-consuming. But it supplied better image quality for biventricular assessment. With feature tracking technique, addition image acquisition (tagging) is not required and it can be easily applied in routine CMR scans with short postprocessing time.

### 4.5. Limitations

There are some limitations to this study. Only a small number of animals are involved. Further, CMR tagging is a gold standard for CMR myocardial strain analysis. A previous clinical study shows that regional CMR-FT may be highly variable compared with CMR tagging [7]. The validation with CMR tagging needs further investigation.

## 5. Conclusions

The analysis of myocardial strain derived from high-resolution conventional cine images using CMR-FT technique provides a highly reproducible method for assessing myocardial contraction in small animal models, especially for global LV longitudinal and circumferential strain assessment. For the right ventricle, the global longitudinal strain shows good to excellent reproducibility of intra- and interobserver and interstudy measurements. The global circumferential strain is variable, with the lowest interstudy reproducibility.

## Figures and Tables

**Table 1:** Demographic, volumetric, and functional characteristics of nexilin knockout heterozygous and wild-type mice. MRI data are issued from the first reading.

Parameters	Heterozygous	Wild-type	*p* value
Study population (*n*)	6	6	—
HR	454 ± 33	393 ± 48	0.07
LVEDV (*μ*l)	81.64 ± 4.56	60.43 ± 8.52	<0.001
LVESV (*μ*l)	38.31 ± 6.31	16.55 ± 3.42	<0.0001
LVSV (*μ*l)	43.34 ± 4.73	43.87 ± 6.10	0.87
LVEF (%)	53.20 ± 6.31	72.71 ± 3.52	<0.0001
CO (ml/min)	16.92 ± 1.66	19.91 ± 3.07	0.06
LVMES (mg)	103.17 ± 13.39	102.50 ± 13.02	0.93
LVMED (mg)	98.67 ± 14.99	98.50 ± 12.44	0.98
RVEDV (*μ*l)	59.89 ± 8.11	56.95 ± 8.40	0.55
RVESV (*μ*l)	19.35 ± 4.21	12.85 ± 3.03	<0.05
RVSV (*μ*l)	40.54 ± 4.46	44.10 ± 5.82	0.26
RVEF (%)	67.95 ± 3.41	77.64 ± 2.79	<0.001

Results are shown as the mean ± SD. HR: heart rate; EDV/ESV: end-diastolic/systolic volume; SV: stroke volume; EF: ejection fraction; CO: cardiac output; LV: left ventricle; RV: right ventricle; LVMED/ES: end-diastolic/systolic left ventricular mass.

**Table 2:** Intraobserver, interobserver, and interstudy reproducibilities for global LV and RV strain assessment.

	Parameter	S1 (%)	S2 (%)	Mean difference	Limits of agreement	ICC (95% CI)	Coefficient of variations
Intraobserver	LLS_LAX_	−15.46 ± 4.51	−15.28 ± 4.17	-0.19	-1.58 to 1.21	0.99 (0.96 to 1.00)	3.36%
	LCS_SAX_	−20.74 ± 3.80	−20.69 ± 3.41	-0.05	-2.56 to 2.46	0.94 (0.81 to 0.98)	4.10%
	LRS_SAX_	30.91 ± 6.48	29.48 ± 4.78	1.43	-4.50 to 7.36	0.84 (0.54 to 0.95)	7.06%
	RLS_LAX_	−27.67 ± 5.44	−27.09 ± 5.57	-0.58	-3.17 to 2.02	0.97 (0.89 to 0.99)	3.92%
	RCS_SAX_	−21.15 ± 2.88	−19.52 ± 2.75	-1.64	-4.06 to 0.79	0.78 (0.02 to 0.95)	7.22%
Interobserver	LLS_LAX_	−15.46 ± 4.51	−15.26 ± 4.15	-0.20	-1.98 to 1.58	0.98 (0.93 to 0.99)	3.51%
	LCS_SAX_	−20.74 ± 3.80	−21.65 ± 3.47	0.91	-2.56 to 4.38	0.86 (0.58 to 0.96)	6.74%
	LRS_SAX_	30.91 ± 6.48	30.54 ± 4.95	0.37	-7.61 to 8.36	0.76 (0.36 to 0.93)	8.58%
	RLS_LAX_	−27.67 ± 5.44	−25.27 ± 6.34	-2.4	-7.57 to 2.79	0.84 (0.30 to 0.96)	11.41%
	RCS_SAX_	−21.15 ± 2.88	−19.47 ± 3.29	-1.68	-6.09 to 2.73	0.65 (0.11 to 0.89)	9.98%
Interstudy	LLS_LAX_	−15.46 ± 4.51	−15.02 ± 4.25	-0.44	-4.26 to 3.37	0.90 (0.71 to 0.97)	8.81%
	LCS_SAX_	−20.74 ± 3.80	−20.41 ± 4.67	-0.33	-6.11 to 5.44	0.77 (0.38 to 0.93)	11.11%
	LRS_SAX_	30.91 ± 6.48	31.49 ± 7.97	-0.57	-15.07 to 13.92	0.50 (0.10 to 0.83)	18.06%
	RLS_LAX_	−27.67 ± 5.44	−25.53 ± 5.77	-2.14	-10.09 to 5.81	0.70 (0.26 to 0.90)	11.99%
	RCS_SAX_	−21.15 ± 2.88	−19.27 ± 3.12	-1.88	-8.29 to 4.52	0.36 (0.14 to 0.74)	12.88%

Results are shown as the mean ± SD. S1 and S2 refer to the two readings among intraobserver, interobserver, and interstudy reproducibilities. LLS_LAX_: left ventricular long-axis longitudinal strain; LRS_LAX_: left ventricular long-axis radial strain; LCS_SAX_: left ventricular short-axis circumferential strain; LRS_SAX_: left ventricular short-axis radial strain; RLS_LAX_: right ventricular long-axis longitudinal strain; RCS_SAX_: right ventricular short-axis circumferential strain; ICC: intraclass correlation coefficient; CI: confidence interval.

**Table 3:** Intraobserver, interobserver, and interstudy reproducibilities for segmental LV and RV strain assessment.

	Parameter	Mean difference	Limits of agreement	ICC (95% CI)	Coefficient of variations
Intraobserver	LLS_LAX_	-0.16	-9.16 to 8.83	0.94 (0.92 to 0.96)	7.41%
	LCS_SAX_	-0.21	-5.98 to 5.57	0.95 (0.94 to 0.96)	14.5%
	LRS_SAX_	1.08	-9.46 to 11.62	0.95 (0.93 to 0.96)	7.6%
	RLS_LAX_	-0.01	-11.96 to 11.99	0.94 (0.91 to 0.96)	5.79%
	RCS_SAX_	-1.51	-9.42 to 6.39	0.78 (0.67 to 0.85)	10.11%
Interobserver	LLS_LAX_	-0.19	-10.06 to 9.68	0.93 (0.90 to 0.96)	19.81%
	LCS_SAX_	1.11	-16.42 to 18.64	0.54 (0.43 to 0.63)	37.4%
	LRS_SAX_	-2.03	-28.66 to 24.60	0.64 (0.55 to 0.72)	42.6%
	RLS_LAX_	-1.74	-13.61 to 10.14	0.95 (0.92 to 0.97)	19.5%
	RCS_SAX_	-1.13	-14.57 to 12.31	0.47 (0.27 to 0.63)	18.35%
Interstudy	LLS_LAX_	-0.05	-16.04 to 15.94	0.80 (0.71 to 0.87)	21.74%
	LCS_SAX_	-0.52	-8.79 to 7.76	0.91 (0.88 to 0.93)	4.4%
	LRS_SAX_	0.18	-18.44 to 18.79	0.84 (0.79 to 0.88)	58.3%
	RLS_LAX_	-0.79	-21.56 to 19.98	0.80 (0.69 to 0.87)	6.5%
	RCS_SAX_	-2.58	-14.35 to 9.19	0.54 (0.36 to 0.69)	15.29%

Results are shown as the mean ± SD. S1 and S2 refer to the two readings among intraobserver, interobserver, and interstudy reproducibilities. LLS_LAX_: left ventricular long-axis longitudinal strain; LRS_LAX_: left ventricular long-axis radial strain; LCS_SAX_: left ventricular short-axis circumferential strain; LRS_SAX_: left ventricular short-axis radial strain; RLS_LAX_: right ventricular long-axis longitudinal strain; RCS_SAX_: right ventricular short-axis circumferential strain; ICC: intraclass correlation coefficient; CI: confidence interval.

**Table 4:** Influence of temporal resolution on feature tracking accuracy of biventricular strain analysis.

Parameter	10 frames	15 frames	20 frames	30 frames	40 frames	60 frames	80 frames
LLS_LAX_	−13.81 ± 2.79^∗∗∗∗^	−17.46 ± 4.55	−17.92 ± 4.62	−17.34 ± 4.55	−17.23 ± 4.20	−17.05 ± 4.24	−16.51 ± 3.87
LCS_SAX_	−14.93 ± 2.37^∗∗∗∗^	−19.78 ± 3.73	−20.32 ± 4.14	−20.35 ± 4.12	−20.08 ± 4.14	−20.07 ± 4.16	−19.56 ± 3.83
LRS_SAX_	28.18 ± 4.61^∗∗∗∗^	34.52 ± 6.37^∗∗∗^	38.03 ± 7.20	39.57 ± 7.34	39.90 ± 7.17	39.24 ± 7.60	39.36 ± 6.73
RLS_LAX_	−22.76 ± 2.29^∗∗∗∗^	−29.40 ± 3.29	−30.30 ± 3.28	−29.70 ± 3.46	−29.75 ± 3.95	−30.05 ± 2.77	−31.06 ± 2.83
RCS_SAX_	−19.61 ± 2.60^∗∗^	−23.55 ± 3.16	−24.77 ± 3.36	−24.74 ± 3.94	−24.82 ± 3.58	−24.67 ± 3.68	−24.92 ± 3.10

Results are shown as the mean ± SD. ^∗^*p* < 0.05, ^∗∗^*p* < 0.01, ^∗∗∗^*p* < 0.001, and ^∗∗∗∗^*p* < 0.0001. LLS_LAX_: left ventricular long-axis longitudinal strain; LCS_SAX_: left ventricular short-axis circumferential strain; LRS_SAX_: left ventricular short-axis radial strain; RLS_LAX_: right ventricular long-axis longitudinal strain; RCS_SAX_: right ventricular short-axis circumferential strain.

## Data Availability

The datasets generated and analyzed are available from the corresponding author in a reasonable request.

## Abbreviations

CMR: Cardiovascular magnetic resonance
RF: Radiofrequency
CMR-FT: CMR feature tracking
Nexn-KO: Nexilin knockout
Het: Heterozygous
WT: Wild-type
b-SSFP: Balanced steady-state free precession
SENSE: Sensitivity encoding
semi-2/4CH: Semi-two/four chamber
SAX: Short axes
EDV: End-diastolic volume
ESV: End-systolic volume
SV: Stroke volume
EF: Ejection fraction
LVMED/LVMES: Left ventricle mass at end-diastole/end-systole
CO: Cardiac output
LV: Left ventricle
RV: Right ventricle
LLS_LAX_: LV long-axis longitudinal strain
LCS_SAX_: LV short-axis circumferential strain
LRS_SAX_: LV short-axis radial strain
RLS_LAX_: RV long-axis longitudinal strain
RCS_SAX_: RV short-axis circumferential strain
SD: Standard deviation
ICC: Intraclass correlation coefficient
CoVs: Coefficient of variations
b-SSFP: Balanced steady-state free precession
STE: Speckle tracking echocardiography
SNR: Signal-to-noise ratio
DCM: Dilated cardiomyopathy
CNR: Contrast-to-noise ratio.

## Supplementary Materials

Supplementary Materials Supplementary Materials S1 quantitative comparison of the SNR_m_ (signal-to-noise ratio in the myocardium), SNR_b_ (signal-to-noise ratio in the LV cavity), and CNR (contrast-to-noise ratio) between the myocardium and LV cavity for different temporal resolutions. ^∗^*p* < 0.05, ^∗∗^*p* < 0.01, ^∗∗∗^*p* < 0.001, and ^∗∗∗∗^*p* < 0.0001. S2: example of qualitative global left ventricle strain curve comparison between different temporal resolutions. A: the myocardial strain segmentation of high-resolution end-diastolic frame of mid-SAX image; B–H: the derived global left ventricle radial and circumferential strain curve for 10, 15, 20, 30, 40, 60, and 80 frames per cardiac cycle. S3: example of qualitative segmental left ventricle strain curve comparison between different temporal resolutions. A: the myocardial strain segmentation of high-resolution end-diastolic frame of mid-SAX image; B–H: the derived segmental left ventricle radial and circumferential strain curve for 10, 15, 20, 30, 40, 60, and 80 frames per cardiac cycle. S4: example of qualitative global right ventricle strain curve comparison between different temporal resolutions. A: the myocardial strain segmentation of high-resolution end-diastolic frame of mid-SAX image; B–H: the derived global right ventricle circumferential strain curve for 10, 15, 20, 30, 40, 60, and 80 frames per cardiac cycle. S5: example of qualitative segmental right ventricle strain curve comparison between different temporal resolutions. A: the myocardial strain segmentation of high-resolution end-diastolic frame of mid-SAX image; B–H: the derived segmental right ventricle radial and circumferential strain curve for 10, 15, 20, 30, 40, 60, and 80 frames per cardiac cycle.
