# Supplementary material for: Quantification of Biventricular Myocardial Strain Using CMR Feature Tracking: Reproducibility in Small Animals
Source: Biomed Res Int. 2021 Jan 22;2021:8492705. doi: 10.1155/2021/8492705 (PMC7847329; doi:10.1155/2021/8492705)
Supplement: Supplementary Materials — S1 quantitative comparison of the SNRm (signal-to-noise ratio in the myocardium), SNRb (signal-to-noise ratio in the LV cavity), and CNR (contrast-to-noise ratio) between the myocardium and LV cavity for different temporal resolutions. ∗p < 0.05, ∗∗p < 0.01, ∗∗∗p < 0.001, and ∗∗∗∗p < 0.0001. S2: example of qualitative global left ventricle strain curve comparison between different temporal resolutions. A: the myocardial strain segmentation of high-resolution end-diastolic frame of mid-SAX image; B–H: the derived global left ventricle radial and circumferential strain curve for 10, 15, 20, 30, 40, 60, and 80 frames per cardiac cycle. S3: example of qualitative segmental left ventricle strain curve comparison between different temporal resolutions. A: the myocardial strain segmentation of high-resolution end-diastolic frame of mid-SAX image; B–H: the derived segmental left ventricle radial and circumferential strain curve for 10, 15, 20, 30, 40, 60, and 80 frames per cardiac cycle. S4: example of qualitative global right ventricle strain curve comparison between different temporal resolutions. A: the myocardial strain segmentation of high-resolution end-diastolic frame of mid-SAX image; B–H: the derived global right ventricle circumferential strain curve for 10, 15, 20, 30, 40, 60, and 80 frames per cardiac cycle. S5: example of qualitative segmental right ventricle strain curve comparison between different temporal resolutions. A: the myocardial strain segmentation of high-resolution end-diastolic frame of mid-SAX image; B–H: the derived segmental right ventricle radial and circumferential strain curve for 10, 15, 20, 30, 40, 60, and 80 frames per cardiac cycle. [file 8492705.f1.docx]

**Supplementary material**

**S1:** Quantitative comparison of the SNR_m_ (signal-to-noise ratio in the myocardium=, SNR_b_ (signal-to-noise ratio in the LV cavity), and CNR (contrast-to-noise ratio) between myocardium and LV cavity for different temporal resolution. *p<0.05, **p<0.01, ***p<0.001, ****p<0.0001.


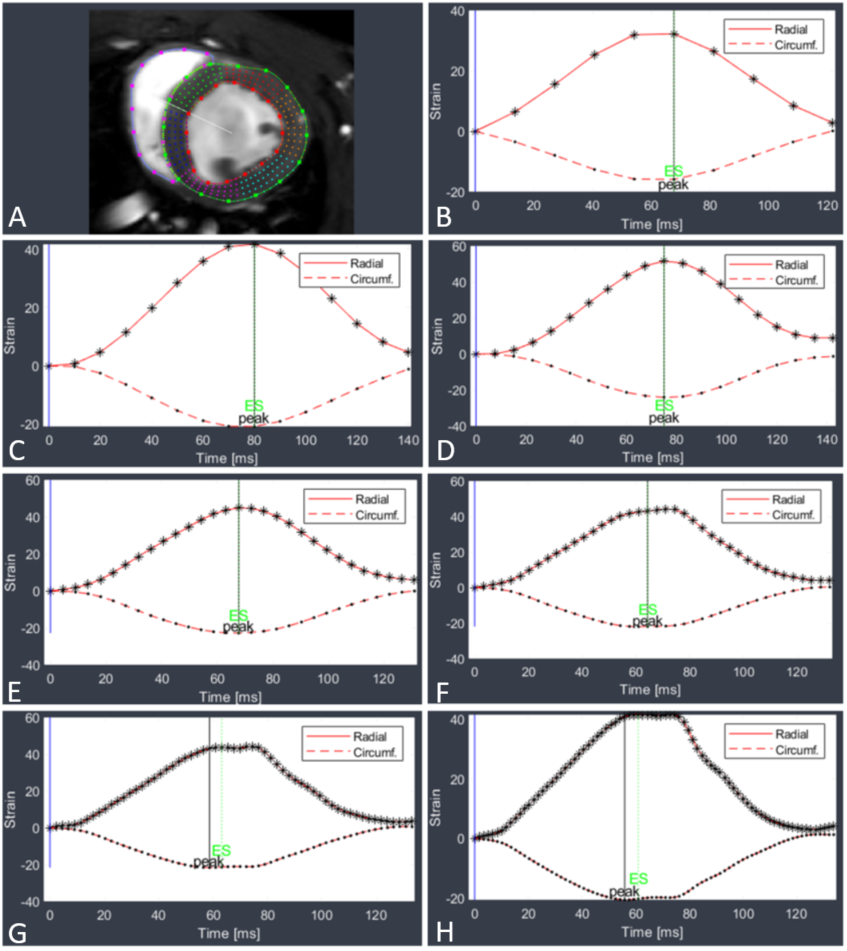


**S2:** Example of qualitative global left ventricle strain curve comparison between different temporal resolutions. A: the myocardial strain segmentation of high resolution end-diastolic frame of mid-SAX image; B – H: the derived global left ventricle radial and circumferential strain curve for 10, 15, 20, 30, 40, 60, 80 frames per cardiac cycle.


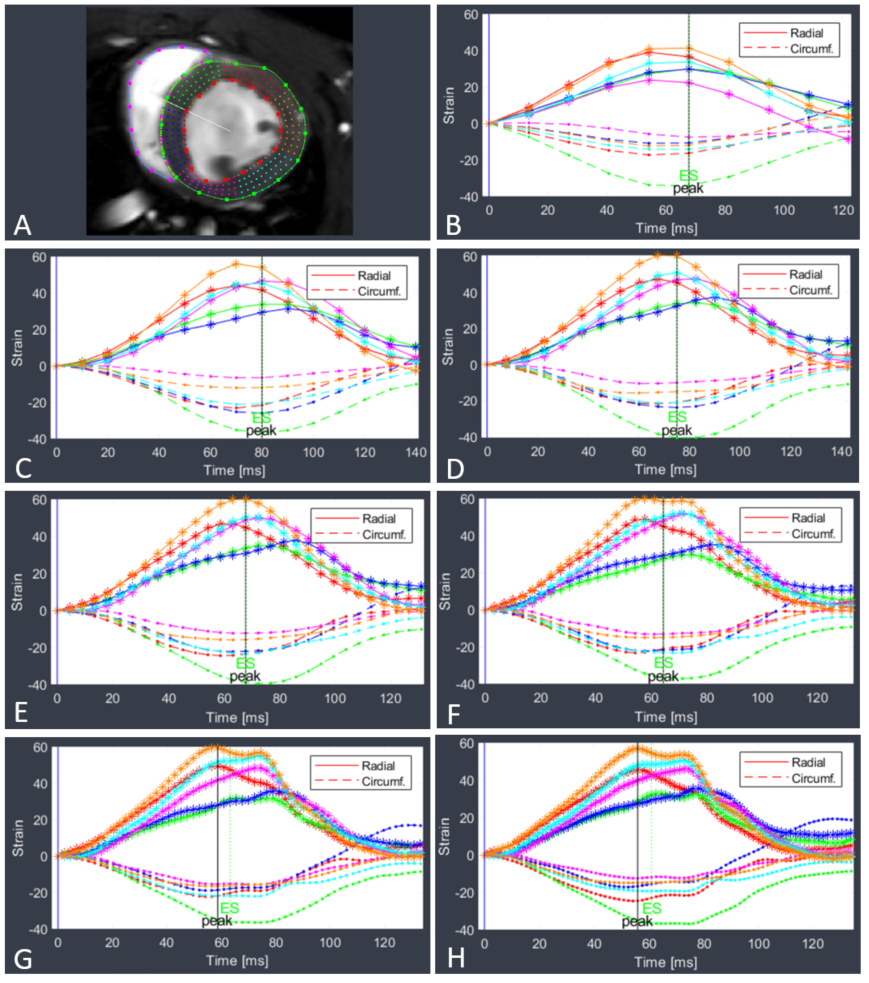


**S3:** Example of qualitative segmental left ventricle strain curve comparison between different temporal resolutions. A: the myocardial strain segmentation of high resolution end-diastolic frame of mid-SAX image; B – H: the derived segmental left ventricle radial and circumferential strain curve for 10, 15, 20, 30, 40, 60, 80 frames per cardiac cycle.


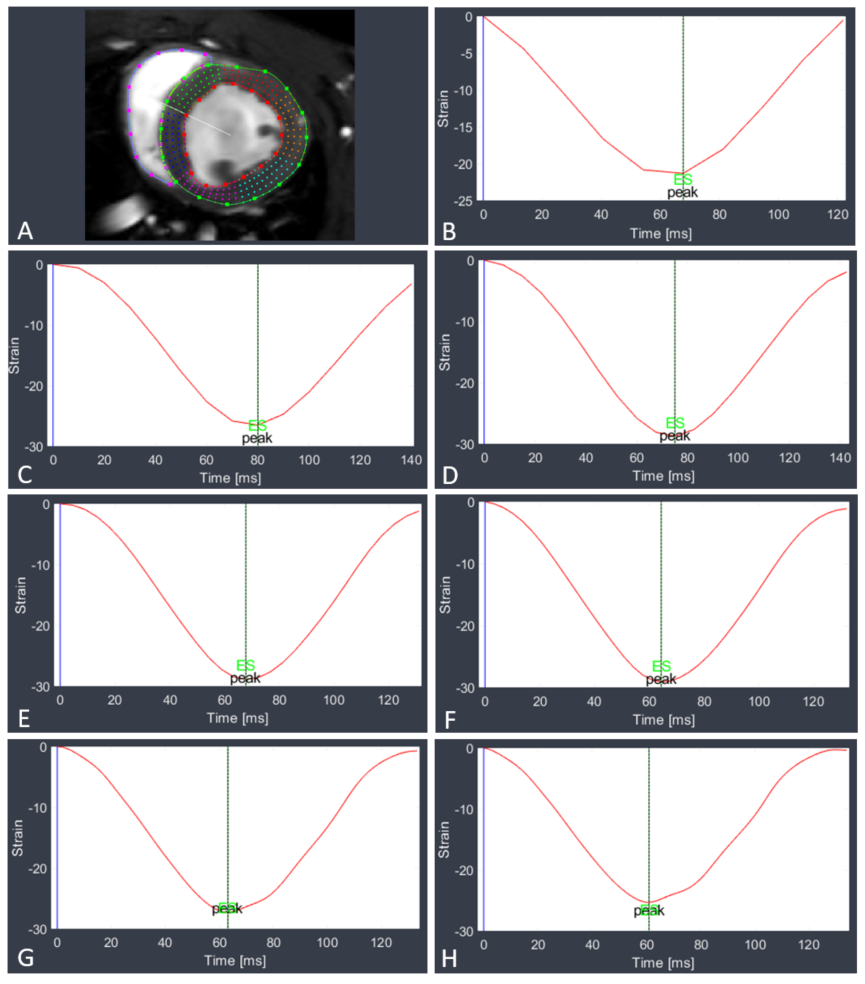


**S4:** Example of qualitative global right ventricle strain curve comparison between different temporal resolutions. A: the myocardial strain segmentation of high resolution end-diastolic frame of mid-SAX image; B – H: the derived global right ventricle circumferential strain curve for 10, 15, 20, 30, 40, 60, 80 frames per cardiac cycle.


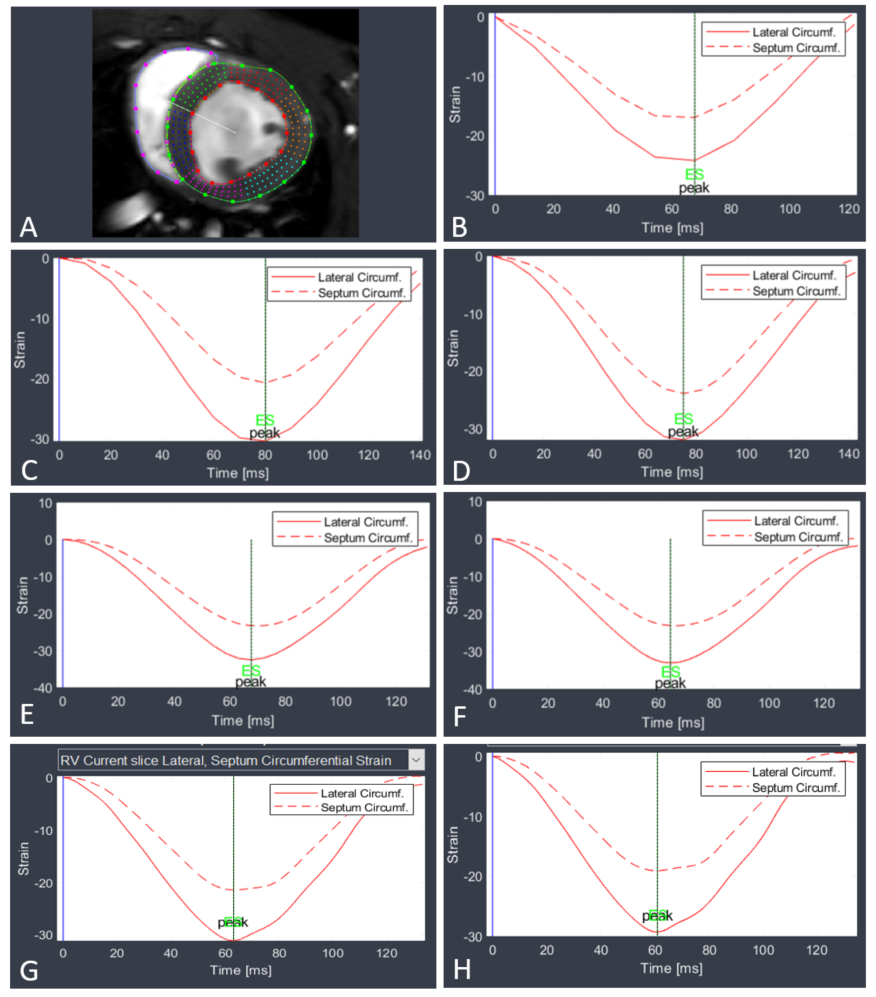


**S5:** Example of qualitative segmental right ventricle strain curve comparison between different temporal resolutions. A: the myocardial strain segmentation of high resolution end-diastolic frame of mid-SAX image; B – H: the derived segmental right ventricle radial and circumferential strain curve for 10, 15, 20, 30, 40, 60, 80 frames per cardiac cycle.
